# The Bone Marrow Microenvironment in Immune-Mediated Inflammatory Diseases: Implications for Mesenchymal Stromal Cell-Based Therapies

**DOI:** 10.1093/stcltm/szad086

**Published:** 2023-12-14

**Authors:** Juliana Redondo, Steven Bailey, Kevin C Kemp, Neil J Scolding, Claire M Rice

**Affiliations:** Translational Health Sciences, Bristol Medical School, University of Bristol, Bristol, UK; Translational Health Sciences, Bristol Medical School, University of Bristol, Bristol, UK; Translational Health Sciences, Bristol Medical School, University of Bristol, Bristol, UK; Translational Health Sciences, Bristol Medical School, University of Bristol, Bristol, UK; Translational Health Sciences, Bristol Medical School, University of Bristol, Bristol, UK; Department of Neurology, Southmead Hospital, North Bristol NHS Trust, Bristol, UK

**Keywords:** cell-based therapy, mesenchymal stromal cells, bone marrow microenvironment, autologous transplantation, immune-mediated inflammatory disorders, multiple sclerosis

## Abstract

Bone marrow (BM)-derived mesenchymal stromal cells (MSCs) are promising candidates for cell-based therapy for several immune-mediated inflammatory diseases (IMIDs) due to their multiplicity of immunomodulatory and reparative properties and favorable safety profile. However, although preclinical data were encouraging, the clinical benefit demonstrated in clinical trials of autologous MSC transplantation in a number of conditions has been less robust. This may be explained by the growing body of evidence pointing to abnormalities of the bone marrow microenvironment in IMIDs, including impaired MSC function. However, it is not currently known whether these abnormalities arise as a cause or consequence of disease, the role they play in disease initiation and/or progression, or whether they themselves are targets for disease modification. Here, we review current knowledge about the function of the BM microenvironment in IMIDs including multiple sclerosis, systemic lupus erythematosus, rheumatoid arthritis, and type I diabetes, focusing on MSCs in particular. We predict that an improved understanding of disease-related changes in the bone marrow microenvironment including the role of MSCs in vivo, will yield new insights into pathophysiology and aid identification of new drug targets and optimization of cell-based therapy in IMIDs.

**Significance Statement**

Cell-based therapy employing autologous mesenchymal stromal cell (MSC) transplantation is a putative treatment for several immune-mediated inflammatory diseases but, in comparison to preclinical studies, the results of clinical trials have been disappointing. This may be explained by emerging evidence demonstrating abnormalities of the bone marrow microenvironment and MSC function in IMIDs. This, in turn, raises important questions about the pathophysiology of the bone marrow microenvironment in IMIDs, including the role of MSCs in the development/maintenance of disease and whether the bone marrow “niche” is a target for disease modification.

## Introduction

Cell-based therapies have generated a great deal of interest as new therapeutic approaches for tissue regeneration and repair. Human bone marrow (BM) mesenchymal stromal cells (MSCs) represent a promising candidate due to their relatively favorable safety profile, low immunogenicity and multiplicity of trophic, immunomodulatory and reparative properties.^1,2^ The therapeutic effects of MSCs rely to a large degree on their capacity to detect cues released at the site of injury and the consequent changes in their “secretome”—the secretion of soluble factors including anti-apoptotic, anti-oxidant, anti-inflammatory, and trophic factors,^3,4^ as well as vesicles containing proteins, intracellular receptors, and genetic material.^4^ Fusion of BM-derived MSCs with host cells has also emerged as a method of delivering functional genes into aged or degenerating cells.^5^

Due to the encouraging therapeutic effects of MSCs in models of disease, putative treatments employing MSCs moved rapidly from pre-clinical studies to clinical trials in a range of disorders, including immune-mediated inflammatory diseases (IMIDs) such as multiple sclerosis (MS), systemic lupus erythematosus (SLE), rheumatoid arthritis (RA), and type I diabetes (T1DM). In preclinical studies, allogeneic MSCs are typically obtained from healthy animals or human subjects. In contrast, clinical trials have largely focused on autologous transplantation. Phase I clinical trials have shown infusion of MSCs is safe and transplant-related serious adverse events are uncommon.^6^ However, the outcomes of randomized controlled clinical trials have been variable and frequently failed to reproduce the often-impressive effects seen in vitro and in disease models. These apparently conflicting results could, at least in part, be explained by differences in trial protocols and factors such as donor/patient age and treatment history.^6^ Furthermore, MSCs are not constitutively anti-inflammatory or immunosuppressive but alter their function in vitro, in response to the environment.^7^ Even more significantly, recent reports highlight MSCs dysfunction and premature senescence in IMIDs (summarized in Table 1). These important factors have, as a rule, not been considered in preclinical studies, but they highlight the importance of considering the host BM microenvironment in disease states and following exposure to immunomodulatory therapies.

**Table 1.** Reported altered phenotype of human MSCs in IMIDs.

IMIDs	Source hMSC	Properties								Ref
		Proliferation	CFU	Senescence	Telomere	Secretome	Immunomodulation	Gene expression	Other	
**MS**	Bone marrow	=	n/a	n/a	n/a	↑ IP-10	= Antiproliferative ability on T cells; = inhibition of DCs	n/a	= immune phenotype	^8^
**MS**	Bone marrow	=	n/a	n/a	n/a	n/a	n/a	n/a	= immune phenotype	^9^
**MS**	Bone marrow	Longer time to reach confluence	n/a	Senescence appearance	n/a	↓ IL-10, TGF	↓ Antiproliferative ability on T cells	618 Differentially expressed genes	n/a	^10^
**MS**	Bone marrow	n/a	n/a	n/a	n/a	↑ IL-6, CCl2, Timp1, IL-8	n/a	n/a	less effective in improving functional recovery in EAE	^11^
**MS**	Bone marrow	↓	↓	↑ βGAL, altered morphology	Shorter	n/a	n/a	n/a	↓ STRO-1	^12^
**MS**	Bone marrow	n/a	n/a	n/a	n/a	↓ Neuroprotection	n/a	n/a	n/a	^13^
**MS**	Bone marrow	n/a	n/a	↑ βGAL after nitric oxide exposure	n/a	↓SOD1, GSTP1	n/a	↓SOD1 and Nrf2 after nitric oxide exposure	↓SOD1, GSTP1, Nrf2 and PGC1α protein expression	^14^
**SLE**	Bone marrow	↓	n/a	↑ βGAL; ↑ cells in G1;	n/a	n/a	↓ Tregs	n/a	↑ P16; ↓CDK4, CDK6, pRB	^15^
**SLE**	Bone marrow	n/a	n/a	↑ Senescence; ↑ apoptotic cells	n/a	n/a	n/a	n/a	↑ Bax, caspase8, Fas and TNFα receptors; ↑ROS	^16^
**SLE**	Bone marrow	n/a	n/a	n/a	n/a	n/a	n/a	1905 differentially expressed genes	Abnormal actin; ↓ cyclin E; activated MAPK pathway	^17^
**SLE**	Bone marrow	↓	↓	↑ βGAL; ↑ cells in G1; ↑ p21 and p53	n/a	n/a	n/a	n/a	Knockdown p21 reversed senescence	^18^
**SLE**	Bone marrow	n/a	n/a	n/a	n/a	n/a	↓ Suppression of B cells	↓ Transcription of CCl2	Lupus MSCs less effective as treatment	^19^
**SLE**	Bone marrow	n/a	n/a	n/a	n/a	n/a	↓ Suppression of T cells	↓ IDO mRNA	n/a	^20^
**SLE**	Bone marrow	n/a	n/a	n/a	n/a	n/a	n/a	n/a	↓ Migration capacity; abnormal cytoskeleton; ↑ROS	^21^
**SLE**	Bone marrow	↓	n/a	↑ βGAL	n/a	↓ IL-10, TGFβ; ↑ IL-6, IL-17	↓ Ratio Treg/Th17	n/a	↑ apoptosis; ↑PTEN	^22^
**SLE**	Bone marrow	↓ Ki67	n/a	↑ βGAL, p53, p16	n/a	↑ IL-6, IL-8, RANTES, GM-CSF	n/a	↓ TGFβ, IDO1, MAVS genes	↑ DNA breaks and damage; ↑MAVs; ↑ROS; silencing MAVS rescued MSC	^23^
**RA**	Bone marrow	↓	↓	↑	Premature loss	= TNFα, IL-1β, IL-6, IL-8, IL-15	n/a	Differentially expressed adhesion, cytoskeleton and cell cycle genes	= immune phenotype	^24^
**RA**	Bone marrow	↓	n/a	↑ p21	n/a	= Cytokine profile	= Antiproliferative ability on PBMC; ↓ inhibition Th17 polarization	↓ mRNA for CCl2	aberrant migration capacity	^25^
**Diabetes** **type 1**	Bone marrow	n/a	n/a	n/a	n/a	n/a	n/a	2978 Differentially expressed genes (↓ CXCL12, VCAM, HGF)	= Morphology, immuno- phenotype, differentiation potential	^26^

**Abbreviations**: Bax, bcl-2-like protein 4; CCl2, chemokine (C-C motif) ligand 2; CDK, cyclin-dependent kinases; CXCL12, C-X-C motif chemokine 12; EAE, experimental autoimmune encephalomyelitis; GM-CSF, granulocyte-macrophage colony-stimulating factor; GSTP1, glutathione S-transferase P 1; HGF, hepatocyte growth factor hMSCs, human mesenchymal stromal cells; IDO, indoleamine 2,3-dioxygenase; IL, interleukin; IMIDs, immune-mediated inflammatory diseases; IP-10, interferon gamma-induced protein 10; MAPK, mitogen-activated protein kinase; MAVS, mitochondrial antiviral signalling protein; MS, multiple sclerosis; n/a, not applicable; Nrf2, nuclear factor erythroid 2-related factor 2; p16, cyclin-dependent kinase inhibitor 2A; p21, cyclin-dependent kinase inhibitor p21; p53, tumor protein p53; PBMC, peripheral blood mononuclear cells; PGC1α, peroxisome proliferator-activated receptor-gamma coactivator 1α; pRB, retinoblastoma protein; PTEN, phosphatase and tensin homolog; RA, rheumatoid arthritis; RANTES, chemokine (C-C motif) ligand 5; ROS, reactive oxygen species; SLE, systemic lupus erythematosus; SOD, superoxide dismutase; STRO-1, stromal precursor antigen 1; TGF, transforming growth factor; Th17, T helper17 cell; Timp1, metallopeptidase inhibitor 1; TNF, tumor necrosis factor; Treg, T regulatory cell; VCAM, vascular cell adhesion protein 1; βGAL, β-galactosidase.

To realize the full therapeutic potential of autologous transplantation of BM-derived cells, including MSCs, it will therefore be important to understand the effect of chronic inflammatory conditions on the BM niche and their influence on MSC function. In turn, this improved knowledge of the underlying pathophysiology may yield insights into biomarker identification and novel treatment approaches.

## MSC Dysfunction in IMIDs

Thorough reviews of bone marrow-derived cell-based therapy are available,^6,8,9^ including those discussing ongoing or concluded clinical trials in MS,^10,12^ SLE,^11,13^ RA,^13,27^ and T1DM.^14,15^ However, examination of the functional properties of MSCs and the BM microenvironment in IMIDs is a relatively nascent field of investigation. To date, the majority of data are available for MS, SLE, RA, and T1DM, which will be the focus of this review (Table 1).

### Multiple Sclerosis

The first systematic exploration of MSC function in MS was published in 2008. Mazzanti et al compared the phenotype, proliferative and differentiation ability, cytokine production, and immunomodulatory activity of MSCs isolated from 10 people with MS (MS-MSCs) and 6 healthy donors (control-MSCs).^16^ They showed that, while MS-MSCs exhibited similar properties to control-MSCs, they over-expressed interferon gamma-induced protein 10, which is known to mediate T-cell recruitment. Previously, we examined MSCs from a small number of people with MS,^17^ and reported they were similar to control-MSCs in the “core” properties of proliferation, mesenchymal differentiation potential, and cell surface antigen expression. However, in subsequent studies involving a larger cohort of people with progressive MS and accounting for effects of age and in vitro passage number, we demonstrated a range of alterations in MS-MSC properties when compared to control-MSCs, including reduced expansion potential, colony forming unit (CFU) capacity, and expression of the early stromal precursor STRO-1, as well as premature senescence and reduced telomere length.^21^ Significantly, the MS-MSC secretome provided reduced neuroprotective potential in comparison to the secretome of control-MSCs.^19^ We also demonstrated that MS-MSCs are more susceptible to nitrosative stress and display decreased activity, expression and secretion of antioxidant molecules, and reduced expression of master regulators of antioxidant responses.^22^ Interestingly, there was a negative correlation between antioxidant response and duration in years of MS progression, raising the possibility that chronic exposure to the disease compromises MSC function. Others have shown that MS-MSCs have a distinct gene expression profile with 618 differentially expressed genes, reduced anti-proliferative effects when cocultured with allogenic T lymphocytes, and decreased secretion of both interleukin (IL)-10 and transforming growth factor (TGF).^20^

In the MS disease model experimental autoimmune encephalomyelitis (EAE), delivery of MSCs isolated from either BM^23^ or adipose tissue^24^ of affected mice lacked the therapeutic efficacy reported in studies using MSCs isolated from unaffected mice, and a similar lack of effect was observed with MSCs isolated from people with MS.^23^ In the latter study, conditioned medium from MS-MSCs was noted to have higher concentrations of pro-inflammatory cytokines compared to conditioned medium prepared from MSCs not exposed to disease conditions.

### Systemic Lupus Erythematosus

Several studies have demonstrated abnormalities in BM-derived MSCs isolated from people with systemic lupus erythematosus (SLE-MSCs), including differential expression of a total of 1905 genes.^25^ Gu et al reported that SLE-MSCs had slower rates of proliferation and increased β-galactosidase (β-GAL) expression consistent with premature senescence.^28^ They also showed that p16^INK4A^, a tumor suppressor that inhibits the cyclin-dependent kinases CDK4 and CDK6, was significantly upregulated in SLE-MSCs. Knockdown of p16^INK4A^ reversed the senescent features of SLE-MSCs. Others have also reported that SLE-MSC are more likely to express features associated with senescence,^29^ as well as apoptosis and aging.^26,30^ Altered functional properties of SLE-MSC that may contribute to pathophysiology include altered migratory capacity,^31^ increased production of reactive oxygen species (ROS),^26,30,31^ and differential cytokine expression,^30^ including pro-inflammatory cytokines.^29^ Of particular interest is the reduced immunomodulatory capacity of SLE-MSCs; this has been reported in the context of failure to suppress B-cell proliferation and differentiation,^32^ as well as T-cell proliferation,^33^ and the latter may be mediated at least in part by reduced SLE-MSC secretion of indoleamine 2,3-dioxygenase (IDO) in response to interferon-γ (IFN-γ).^33^

### Rheumatoid Arthritis

Kastrinaki et al^34^ investigated the molecular and proteomic profile of MSCs isolated from people with RA-MSCs, together with an examination of their functional properties in vitro. Similar to our findings in MS,^21^ they reported that RA-MSCs have decreased CFU and proliferative capacity, as well as premature telomere length loss. Transcriptome analysis revealed differential expression of genes related to cell adhesion processes and cell cycle progression. However, no differences in differentiation potential, immunomodulation, and cytokine production between RA-MSCs and control-MSCs were identified. Others have since replicated the finding of decreased proliferation potential of RA-MSCs and explored additional functional properties reporting that, although RA-MSCs appeared to be indistinguishable from control-MSCs in suppressing peripheral blood mononuclear cell proliferation and inducing polarization of regulatory T (Treg) cells, inhibition of T-helper 17 (Th17) cell polarization and aberrant migration capacity were noted.^35^

### Type 1 Diabetes

MSCs isolated from people with T1DM (T1DM-MSC) have been less extensively studied, but some concerns regarding the therapeutic potential of autologous MSC cell-based therapy for diabetes mellitus have also emerged.^14,36^ Notably, a microarray study comparing MSC isolated from healthy individuals and people with T1DM revealed differential expression of almost 3000 genes in the T1DM-MSC transcriptional profile, including adhesion proteins, immune mediators, chemotaxis-related molecules, and growth factors.^37^

## Bone Narrow Microenvironment in IMIDs

The BM niche is a highly complex microenvironment in which stromal cells support hematopoietic stem cells (HSCs), orchestrating a delicate balance of quiescence, self-renewal, differentiation, and mobilization.^38^

The lack of single, specific markers has limited the identification of BM-derived stromal cell populations, but recent improvements have facilitated closer interrogation of the role of these cells in vivo. Indeed, MSCs are now well-recognized as key players in the complex niche network of cell-to-cell communication. Given the abnormalities identified in BM-derived MSCs in IMIDs and the key role of the BM in support of HSC proliferation and differentiation, further examination of the BM microenvironment in IMIDs and its role in disease initiation and progression are timely.

### Multiple Sclerosis

The BM microenvironment has been under-investigated in MS and relatively limited information is available regarding its structure and (dys)function. Carrai et al analyzed BM trephines from people with MS undergoing autologous hematopoietic stem cell transplantation; compared to control subjects, no difference in lymphocytes was reported, but there was reduced cellularity in the MS trephines with a trend toward increased fibrosis and reduced matrix metalloproteinase 9 expression.^39^ We replicated the finding of relatively low overall cellularity in the BM microenvironment in samples isolated from people with progressive MS but also noted infiltration with both B and T cells, as well as the presence of T-cell nodules.^21^ The myeloid:erythroid ratio was normal, and all specimens were negative for the tumor suppressor P53 and markers of fibrosis. However, reduced Ki67 expression was in keeping with decreased proliferation. We also identified spindle-shaped vimentin + cells expressing the cyclin-dependent kinase inhibitor p16 in MS marrow, which we interpreted as being indicative of altered stromal cell function in MS marrow.

### Systemic Lupus Erythematosus

Immune cytopenias are associated with SLE. As in MS, the BM has been reported to be relatively hypocellular in SLE, although plasmacytosis,^40,41^ together with increased apoptotic bodies and caspase 3 positive staining,^42^ has also been observed. Others have reported a variety of histopathologic findings including BM necrosis, stromal alterations, hypocellularity, dyspoiesis (abnormal formation of blood cells), and distortion of normal BM architecture, characterized by the presence of abnormal localization of immature precursor aggregates.^43^ Reports from the first French registry of cases of BM abnormalities in SLE have shown that BM fibrosis and pure red cell aplasia are the most frequent BM manifestations, with variable global marrow cellularity.^44^

### Rheumatoid Arthritis

A growing body of evidence suggests that the BM is actively involved in RA. A variety of BM morphological, immunophenotypic, and functional abnormalities have been detected, including increased numbers of mononuclear cells, elevated levels of IL-6 and IL-8 and accumulation of lymphoid aggregates in follicle-like structures (reviewed by^45^). Interestingly, a study characterizing BM hematopoietic progenitor cells in 16 refractory RA patients who were candidates for autologous stem cell transplantation, identified risk factors for impaired mobilization and engraftment.^46^ The authors observed alterations in RA bone marrow including markedly reduced cellularity, lower CD34 + cells and higher apoptotic index in myeloid precursors with respect to healthy controls. Studies have also explored subchondral BM in RA. This fat-rich compartment is in direct contact with the joint space and may be replaced in RA by a vascularized, cell-rich inflammatory tissue characterized by macrophages, plasma cells, and aggregates of T and B cells.^47,48^ This suggests an important pathway for the development of RA-related bone damage: starting from an inflammatory process within BM, leading to recruitment and activation of osteoclasts and ultimately bone erosion.

### Type 1 Diabetes

It has recently been recognized that diabetes induces profound remodeling of the BM niche in mice, rats, and humans, including small vessel disease, neuropathy, and impaired stem cell mobilization (reviewed^49^). In mice with T1DM, the BM has also been reported to have reduced numbers of osteoblasts and higher concentrations of proinflammatory cytokines with defective homing of BM-derived progenitor cells.^49^ Few comparative studies of the BM niche in people with T1DM are available, although one reported extensive fatty infiltration, microvascular rarefaction, decreased numbers of hemopoietic progenitor cells, and increased pro-apoptotic factors on CD34^+^ cells.^50^

## Mechanisms Underlying Altered BM and MSC Function in IMIDs

**Figure 1.**
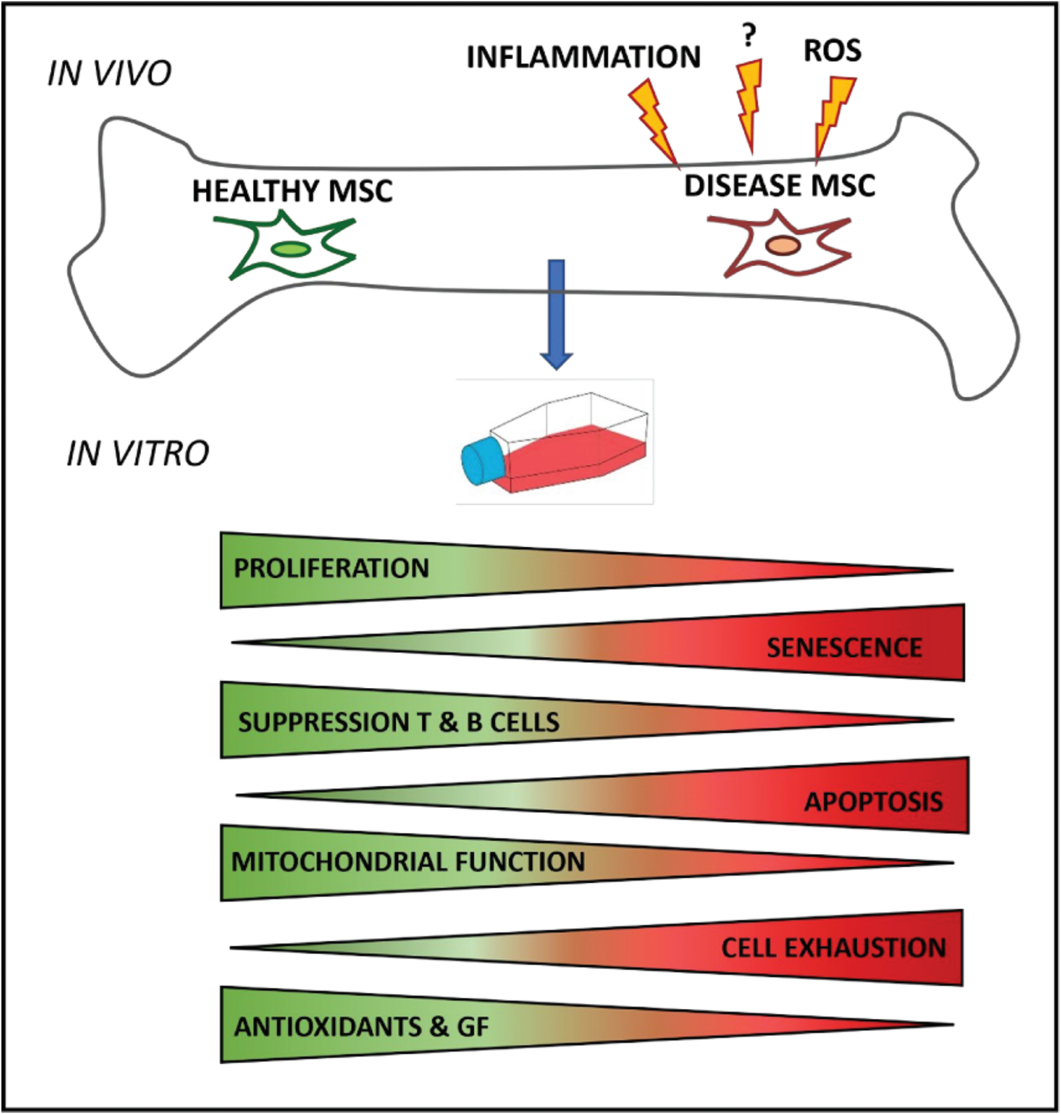
MSCs from patients with immune-mediated inflammatory diseases demonstrate a range of abnormalities in vitro. Abbreviations: GF, grow factors; MSC, mesenchymal stromal cells; ROS, reactive oxidative stress.

**Figure 2.**
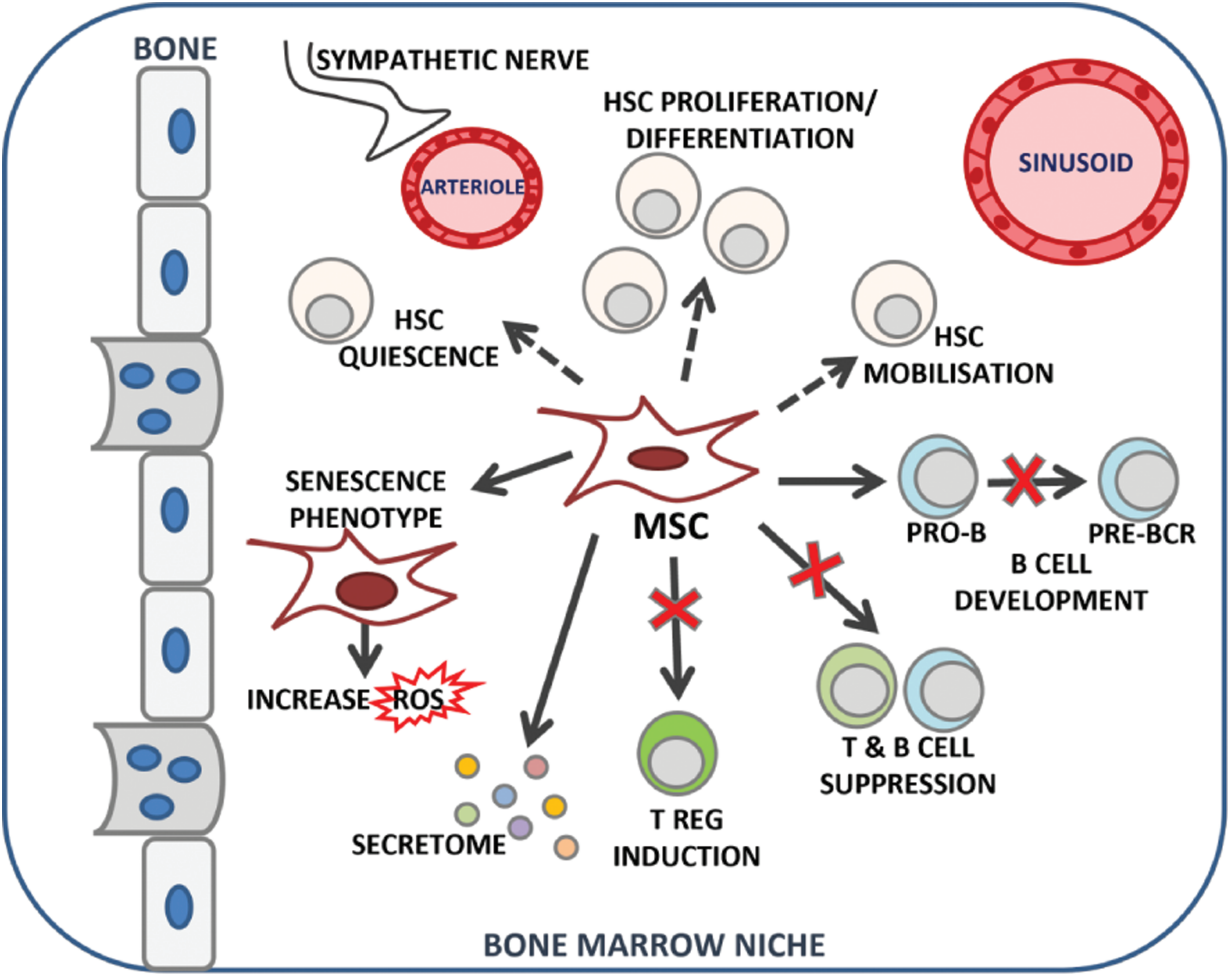
In the bone marrow niche, several mesenchymal stromal cell (MSC) properties can be affected in immune-mediated inflammatory diseases. MSCS can display premature senescence; their role in supporting HSC function regulating quiescence, self-renewal, differentiation, and mobilization can be altered; they can have impaired immunomodulatory capacities, and drive abnormal B-cell receptor development and have alterations in their secretome. Abbreviations: BCR, B-cell receptor HSC, hematopoietic stem cell; MSC, mesenchymal stromal cell; ROS, reactive oxidative stress; T reg, T regulatory cell.

It has long been appreciated that MSC isolated from “healthy” donors of advanced age show reduced proliferative potential accompanied by increased markers of oxidative stress and senescence^51,52^ (Fig. 1). These changes are also reflected in the reduced therapeutic capacity of aged MSCs in animal models of disease.^53,54^ IMIDs may produce similar changes in MSC by augmenting common pathways involved in “normal” in vivo aging, leading to premature senescence and reduced proliferative potential in the MSC pool. This hypothesis overlaps with the concept of “inflamm-aging,” which purports that aging promotes chronic low-grade inflammation, accelerating age-related diseases.^55^ In IMIDs, a pro-inflammatory BM microenvironment could therefore contribute to the observed dysfunction of MSCs. Specific disease-related systemic cytokines and chemokines could be influential via their remote effects on the BM compartment, including cellular proliferation, differentiation, peripheral mobilization, and inflammatory polarization (Fig. 2), including via epigenetic regulation.^56^

Numerous mechanisms have been proposed for stem cell aging, as reviewed by Oh et al^57^ and include: increased oxidative stress and impaired antioxidant defenses, leading to accelerated telomere loss, the accumulation of DNA damage, and premature replicative or stress-induced senescence; mitochondrial dysfunction, leading to increased oxidative stress and impaired bioenergetics; impaired proteasomal activity or autophagy, leading to loss of cellular homeostasis and premature senescence or apoptosis; and epigenetic changes, particularly those associated with senescence. In addition, changes in the extracellular matrix^58^ or the relative proportions of cell populations in the BM could influence MSC proliferation and function.

Alternatively, replicative senescence may occur due to pro-inflammatory signals, which drive the proliferation and mobilization of stem cells to the point of stem cell “exhaustion”^59^—in support of this theory, peripheral mobilization of BM-resident MSCs has been described in one study of EAE, alongside a transient decrease in the bone marrow-resident MSC population.^60^

“Bystander senescence” could also occur in MSCs as a response to the senescence of other cell populations in the BM compartment, highlighting the fact that the response of MSCs to the inflammatory microenvironment cannot be considered in isolation. Progressive impairment of antioxidant defenses, trophic factor expression, and immunosuppressive capacity may reinforce the shift toward higher levels of cellular stress and senescence in the BM.

An alternative hypothesis would purport a genetic link between autoimmune disorders and MSC dysfunction,^61^ but evidence for this is lacking. In fact, it would seem to be relatively unlikely, given that MSC dysfunction is common to a range of IMIDs and genetic backgrounds.

## Strategies to Reverse IMID-Associated MSC Dysfunction and Abnormalities of the BM Microenvironment

Taking into consideration the plethora of mechanisms that may be involved in MSC dysfunction, restoration of MSC function may require multiple approaches and/or a single intervention that acts upon a number of cellular pathways. Approaches may directly target MSC function (for instance, enhancing viability, engraftment, proliferation, migration, and/or the secretion of bioactive molecules) or be directed toward the BM niche as a whole. To date, anti-oxidative, anti-aging, and anti-senescence approaches have been explored (Table 2).

**Table 2.** Strategies to reverse BM cell dysfunction in IMIDs.

Strategies	Method	Ref
Anti-oxidative	SIRT 3 overexpression	^62^
	SOD 3 overexpression	^63^
	Nrf2 overexpression	^64^
	Enhance telomerase activity	^65^
	Regulation by coding and non-coding RNAs	^66^
Anti-aging	Upregulation of SIRT 3	^67^;^68^
	Enhance telomerase activity	^69^
	Autophagy modulation	^70^
	Epigenetic rejuvenation	^71,72^
Anti-senescence	Overexpression of SIRT 1	^73^
	Modulation by non-coding RNAs	^66^
	Lowering levels of oxidative stress	See “Anti-oxidative”
	Resveratrol treatment	^74^
In vitro pre-conditioning	Preconditioning strategies to improve therapeutic efficacy	^75^
Combination therapy	Transplantation of MSC and SOD 1	^76^
	Transplantation of MSC and Resveratrol [see text]	^18^

Since oxidative stress, aging and senescence are major players in MSC dysfunction, strategies to improve any one of these factors could have beneficial effects. For instance, strategies to lower the increased levels of ROS that occur in senescence could help ameliorate the effects of premature cell aging.

Overexpression of antioxidants, transcription factors, cytokines, and growth factors, mediated through natural or synthetic compounds or genetic manipulation, may represent a potential approach to increase the therapeutic efficacy of MSC. Overexpression of members of the sirtuin (SIRT) family, including SIRT1 and SIRT3, can reduce oxidative stress and senescence. Both have deacetylase activity, and are reduced during aging; SIRT3 is of particular interest due to its mitochondrial localization and capacity to promote the transcription of oxidative stress response genes,^77^ while SIRT1 plays a pivotal role in reducing apoptosis, inflammation, and senescence.^78,79^ SIRT3 levels in MSC are decreased under oxidative stress and this reduction is more prominent in MSC derived from older patients.^62^ Conversely, SIRT3 overexpression has been shown to improve MSC resistance to ROS exposure.

Overexpression of superoxide dismutase (SOD) 3 in MSC (SOD3-MSC) has been evaluated^63^: SOD3-MSC exhibits a reduction in intracellular ROS levels, greater survival, attenuation of the negative effects caused by serum deprivation, and increased autophagy, implying that SOD3-MSC may have therapeutic potential. Other approaches could include upregulation of expression of transcription factors such as the nuclear factor erythroid 2-related factor 2 (Nrf2), which controls an array of antioxidant genes; or peroxisome proliferator-activated receptor-gamma coactivator 1α (PGC1α), a master regulator of mitochondrial biogenesis, energy metabolism and induction of antioxidant programs.

Epigenetic dysregulation is a hallmark of aging. DNA methylation changes occur in aging and in the replicative senescence of MSCs. Specific CpG islands become differentially methylated in MSCs upon long-term culture^80,81^ and an “Epigenetic-Senescence-Signature” has been described for the prediction of MSC aging in vitro; this signature could therefore be used for quality control of therapeutic cell preparations.^82^ Since epigenetic changes can be reversed,^71,72^ manipulation of the epigenome may be a further strategy to reprogram old/dysfunctional cells to become “youthful,” with greater therapeutic potential.

Small non-coding RNAs, in particular microRNAs (miRNAs) that act by silencing the expression of specific genes, represent potential sensors of oxidative stress and senescence. miRNAs can induce a senescence phenotype, influence cell cycle progression, help DNA repair, and promote the production of ROS and inflammatory cytokines.^83^ MSCs isolated from old mice have elevated expression of miR-195, which targets the telomerase reverse transcriptase (Tert) gene; and abrogation of miR-195 expression in aged MSCs induced marked Tert activation, with telomere re-lengthening and reduced senescence.^69^ An extensive summary of miRNAs involved in aging provides potential targets for boosting the therapeutic capabilities of stem cells.^83^

Resveratrol is a plant-derived polyphenol with a range of anti-oxidant, anti-inflammatory, anti-aging, and anti-carcinogenic properties. It acts through multiple pathways, including activation of SIRT 1, regulation of microRNAs, and inhibition of NF-kB activation.^84^ Combined administration of resveratrol and BM-MSC in EAE mice leads to the reduction in both disease severity and CNS inflammatory cell infiltration, with increased levels of anti-inflammatory cytokines compared to single treatment with resveratrol or MSCs alone, demonstrating a potential novel protocol for MSC-based therapy.^18^

Extensive research indicates that in vitro pre-conditioning of MSC can enhance their regenerative capacity. In experimental paradigms, pre-treatments with trophic factors (cytokines, chemokines, and hormones), serum deprivation, modulation of oxygen concentration (hypoxia and anoxia), 3D cultures, and pharmacological agents have all shown therapeutic efficacy.^75^ Very few of these approaches have, however, been explored in human clinical studies.

## Clinical Implications and Conclusion

Despite preclinical promise, relatively few randomized controlled clinical trials utilizing MSC transplantation in IMIDs have demonstrated significant clinical benefit for example, ‘MEsenchymal StEm cells for Multiple Sclerosis’ (MESEMS; NCT01854957) failed to meet its primary endpoint.^85^ This may be explained, at least partially, by the effects of the disease environment compromising cell function, although a causative link between dysfunctional MSC and disease initiation and/or progression remains unproven. Indeed, it is unclear whether altered MSC function is a primary or secondary consequence of IMIDs: does an inflammatory environment alter MSC function or do malfunctioning MSCs trigger disease? What is clear is that MSC dysfunction in IMIDs has several common elements: low proliferation, increased senescence, altered secretome, and gene expression profile. Whatever its role in disease initiation, functional compromise of the BM niche is likely to amplify and sustain the disease process; MSC are crucial for HSC maintenance, B-cell receptor development, and T-cell suppression within the BM, and the systemic inflammatory milieu alters the paracrine properties of MSCs and their interactions with HSC/T/B cells in the niche. MSCs from other tissues, including the human placenta and umbilical cord, could represent an alternative MSC source for cell therapy due to their relative accessibility. However, care will need to be taken to assess their functional properties as differences have been reported for example, in cytokine profile^86^ and gene expression profile.^87^

It is evident that multiple challenges must be addressed before the full therapeutic potential of autologous BM-derived cell therapy for IMIDs can be optimized. Given the multiplicity of MSC roles in vivo and potential specificity of disease-related impairments, multiple disease-specific parameters are likely to require evaluation. This will include the determination of the optimal treatment schedule, including timing and number of treatments. An evaluation of the functional effectiveness of the cell source will be critical, particularly for autologous cell transplantation, and identification of biomarkers and assays to predict MSC clinical effectiveness would be advantageous. However, this is likely to require assessment of multiple cell parameters, including for example, surface marker expression, differentiation potential, telomere length, senescence status (including epigenetic signature^82^) secretome composition, and functional assessment of immunomodulatory properties.^88^ Others have suggested that an assessment of the donor’s idiomatic immune status at the time of treatment may be required to predict treatment response.^8^ Further, additional refinements could include combination therapies for example, with immunomodulatory medications or senotherapeutic agents. Overall, we suggest that an improved understanding of MSC function and the BM niche, as well as the interplay between them, will undoubtedly contribute to more complete knowledge of IMID pathophysiology, and identification of novel treatments for IMIDs.

## Data Availability

No new data were generated or analyzed in support of this research.
